# Animal Species Authentication in Dairy Products

**DOI:** 10.3390/foods11081124

**Published:** 2022-04-13

**Authors:** Isabel Mafra, Mónica Honrado, Joana S. Amaral

**Affiliations:** 1 REQUIMTE-LAQV, Faculdade de Farmácia, Universidade do Porto, 4050-313 Porto, Portugal; 2 CIMO, Instituto Politécnico de Bragança, 5300-253 Bragança, Portugal; monica-honrado@hotmail.com

**Keywords:** milk, dairy products, authenticity, analytical methods, adulteration

## Abstract

Milk is one of the most important nutritious foods, widely consumed worldwide, either in its natural form or via dairy products. Currently, several economic, health and ethical issues emphasize the need for a more frequent and rigorous quality control of dairy products and the importance of detecting adulterations in these products. For this reason, several conventional and advanced techniques have been proposed, aiming at detecting and quantifying eventual adulterations, preferentially in a rapid, cost-effective, easy to implement, sensitive and specific way. They have relied mostly on electrophoretic, chromatographic and immunoenzymatic techniques. More recently, mass spectrometry, spectroscopic methods (near infrared (NIR), mid infrared (MIR), nuclear magnetic resonance (NMR) and front face fluorescence coupled to chemometrics), DNA analysis (real-time PCR, high-resolution melting analysis, next generation sequencing and droplet digital PCR) and biosensors have been advanced as innovative tools for dairy product authentication. Milk substitution from high-valued species with lower-cost bovine milk is one of the most frequent adulteration practices. Therefore, this review intends to describe the most relevant developments regarding the current and advanced analytical methodologies applied to species authentication of milk and dairy products.

## 1. Introduction

In recent years, food authenticity has been considered as a core concern regarding the safety and quality control of food, with several regulatory agencies around the world increasingly devoting resources to this issue [1,2]. According to a 2013 report from the European Parliament, milk was ranked as one of the four ingredients/foods considered as the most common targets for economically motivated adulteration [3]. Milk and dairy products are highly nutritive foods, largely consumed by the general population, which play an important role in the diet of particular groups of consumers, namely children and pregnant women. Due to its high demand and value, frauds in the dairy industry have become a widespread problem, but also a real concern for many consumers and authorities, especially after the melamine scandal [4,5,6,7,8]. As for other food commodities, the labeling of dairy products is a key issue because the declared information must match the characteristics of the product, particularly in what concerns the used ingredients and production technology [6]. In dairy products, the detection of mislabeled and/or sub-standard products is of utmost importance for both economic and public health reasons [4,5,6,7]. Additionally, the introduction of milk from non-declared species might have health risks regarding the presence of allergens [9], as well as ethical implications due to religious practices or personal choices that avoid ingesting milk from certain species. Therefore, over the last decade, several methodologies have been proposed for the evaluation of quality and authenticity of dairy products aiming at consumer’s protection, as well as promoting fair competition and general confidence in the sector.

Several types of frauds have been reported to occur in the dairy industry, including the substitution of milk fat and/or milk protein, the substitution of milk from one species with a lower valued one, dilution with water, the addition of fillers, the addition of preservatives, the addition of whey rennet, the application of undeclared processing methods and mislabeling regarding the geographical origin [4,5,6,8]. Among them, the species of origin is one of the most common frauds, namely the substitution of highly valued milks (such as sheep’s, goat’s or buffalo’s) with cow’s milk, to reduce production costs and increase profits [5,8]. This fraudulent practice is frequently associated with seasonal oscillations and a lower production yield of ovine, caprine and bubaline (or more exotic species such as camel or donkey), which raises their economic value. The identification of animal species is also particularly important in the case of high-priced traditional products, such as cheeses labeled with the European Union (EU) logos of protected designation of origin (PDO), protected geographical indication (PGI) or traditional specialty guaranteed (TSG) [5,8]. Currently, in the category of cheeses, there are 260 registered products in the EU geographical indications register database (eAmbrosia), having either the logo of PDO (199), PGI (54) or TSG (7). From the 22 countries of origin, Italy has the highest number of registered cheeses (56), followed by France (55) and Spain (30). Several of these products, besides requiring the use of milk from specific animal species, also specify the animal breed. For example, the Spanish Manchego cheese must be produced from sheep’s milk of the Manchega breed and the Portuguese Terrincho cheese produced from sheep’s milk of the Churra da Terra Quente breed. Since PDO, PGI or TSG cheeses generally command higher prices than other similar products without such labels, they are potential adulteration targets by the substitution of milk from the specified species (or breeds) with others. This has been a driving force for the development of novel methodologies that allow the specific identification of the animal species, but also their quantitative determination in dairy products.

**Figure 1:**
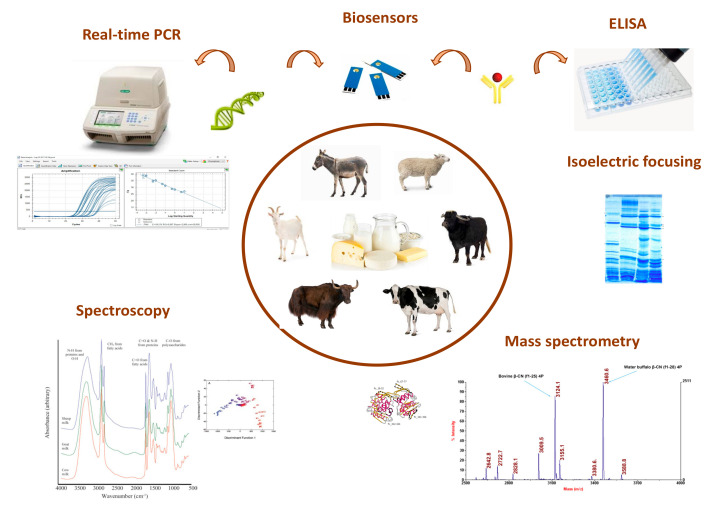
Graphical representation of analytical methods used for the authentication of animal species in dairy products. Adapted from [16,72]. Reprinted from [16,72] with permission from Elsevier.

Considering that species substitution is one of the most relevant authenticity issues in milk and dairy products, this review intends to provide an updated overview referring to the most relevant and recent analytical advances for species authentication in dairy products (Figure 1).

## 2. Protein-Based Methods

Protein-based techniques, including electrophoresis, chromatography and immunochemical assays, are considered current methodologies for assessing the authenticity of dairy products [5]. They are generally considered fast, high throughput and cost-effective, being suitable approaches for the analysis of animal species in raw milk. However, when applied to processed foods, their reliability might be compromised due to protein denaturation and consequent epitope modification, disabling the immunorecognition of proteins. In recent years, the developments of mass spectrometry (MS) platforms for protein analysis, characterization and quantification have provided alternative approaches that rely on marker peptides instead of whole proteins, being suitable alternatives to analyze processed products [10]. Nonetheless, MS methods require costly equipment and specialized personnel. Table 1 and Table 2 present the summarized information on the reported protein-based methods applied to species authentication in dairy products.

### 2.1. Electrophoretic Techniques

Different works using electrophoretic techniques have been reported so far for the detection of milk adulteration, including the use of polyacrylamide gel electrophoresis (PAGE) or, most frequently, the use of isoelectric focusing (IEF) (Table 1). Although PAGE is generally effective, its main limitation concerns the complex band pattern obtained, with frequent overlap of bands that can lead to an equivocal interpretation of results. Pesic et al. [11] suggested the use of a native PAGE electrophoresis for the qualitative and quantitative analysis of bovine adulteration in ovine or caprine milk based on bovine β-lactoglobulins (β-LG) and α-lactalbumins (α-LA). This method was considered a fast and convenient alternative for the detection and estimation of milk adulteration. However, its application is limited to fresh milk mixtures since heat processing and pH can cause the denaturation of whey proteins, with β-lactoglobulins being remarkably affected particularly by severe heat treatments including ultra-high temperature (UHT). 

A similar approach consisting of isoelectric focusing (IEF) of γ-caseins, namely γ2-and γ3-caseins obtained by plasminolysis of β-casein, is currently the reference method in the EU for the determination of cow’s milk caseins in ovine, caprine, and water buffalo cheeses [5,7,12,13,14]. In this method, the samples should be analyzed together with reference standards containing 0% and 1% cows’ milk, being considered positive if both bovine γ2-and γ3-caseins, or the corresponding peak area ratios, are equal to or greater than the level of the 1% reference standard [14]. The method can be used for detecting either raw or heat-treated cow’s milk and caseinate in fresh or ripened cheeses made of ewes’, goats’ and buffalos’ milk or their mixtures, though not being suitable for the detection of milk and cheese adulteration by heat-treated bovine whey protein concentrates [14]. It is also not adequate for species quantification, especially in ternary mixtures due to the similarities between some species, such as ovine and caprine [7,12,15]. In fact, the reference method fails in detecting goat’s milk in sheep’s cheese and milk. Additionally, other works demonstrated that the evaluation of cow’s milk casein in water buffalo cheese by IEF is sometimes uncertain due to the presence of interfering co-migrating bands that can result in false positives [12,15,16]. Recently, Caira et al. [16] used a proteomic approach to demonstrate that this false positive result was due to the water buffalo fragment β-casein(f100-209), which was also formed after plasminolysis of buffalo’s milk or dairy products and co-migrates in IEF with bovine γ2-casein. To avoid false positives due to a water buffalo casein band with an isoelectric point similar to that of bovine γ2-casein, Addeo et al. [15] proposed the use of IEF coupled to immunoblotting to detect the presence of cow’s milk in water buffalo cheeses. In this study, antipeptide antibodies were raised against three sequence stretches of bovine γ2-casein, with one of them, namely anti-β-casein-(106-110), showing to be highly specific for bovine proteins. The methodology proved to be successful in evaluating the authenticity of pure water buffalo milk and cheeses, with a limit of detection up to 0.25% bovine milk (*v/v*), which was lower than that described by the EU reference method (1%).

Capillary electrophoresis (CE) has been suggested as an alternative to gel electrophoresis-based methods for the authenticity assessment of dairy products because of its higher resolution power, low operation cost and high throughput [4,17] (Table 1). Somma et al. [17] compared the efficiency of ultra-thin-layer IEF with capillary isoelectric focusing (cIEF) applied to the separation and identification of the main peptides arising from the hydrolysis of water buffalo and bovine β-caseins. Additionally, cIEF was used in combination with mass spectrometry for structural confirmation of the separated peptides. cIEF proved to be faster and more convenient because it does not require gel staining, though the cow-specific markers were only detectable at 5% cow’s milk addition in water buffalo’s milk, a value well above the sensitivity of the IEF method (0.5%). Nevertheless, both methods could be useful for detecting the fraudulent addition of cow’s milk to buffalo’s milk, which is very important in the production of Mozarella di Bufala cheese. More recently, Trimboli et al. [18] proposed the use of a routine CE method for human blood and urine protein analysis as a tool to authenticate ewe’s skimmed milk. The method was based on the separation of skimmed milk proteins and the use of a characteristic peak for ewe’s milk quantification in ovine/bovine skimmed milk mixtures, allowing us to detect a minimum amount of 5% of added cow’s milk with good linearity, precision and accuracy. A similar approach, using a routine CE method for blood analysis, was also attempted for detecting as low as 1% cow’s milk in buffalo’s milk and predicting the amount of fraudulently added milk by exploiting cow’s α-lactalbumin as a marker of adulteration [19]. Although most works dealing with the application of CE have been applied to milk mixtures, its use for the successful identification of animal species in cheese samples has also been demonstrated [20,21].

### 2.2. Immunochemical Techniques

Immunochemical methods are often used in the food industry for the qualitative and quantitative detection of food components and/or contaminants, being applied since the early 1980s to answer to the analytical demands in the dairy industry [7,13,22]. Essentially, an immunochemical assay consists of the reaction of an antigen with a specific antibody [13]. Therefore, immunochemical techniques provide highly specific and sensitive methods, being applied to a variety of complex food products. Compared with electrophoretic and chromatographic techniques, they are considered generally simpler, of lower cost, more sensitive and specific [7,13].

Enzyme-linked immunosorbent assay (ELISA) is the immunochemical technique most frequently used in dairy product analysis with diverse formats, including direct, indirect, sandwich and competitive, being applied to detect whey proteins and caseins (Table 1). ELISA are frequently used in the analysis of milk and dairy products because of their easy application in routine analysis, low-cost, speed and sensitivity. However, the selected antisera influences the specificity and sensitivity of the method, thus requiring specific antibodies capable of differentiating species, without providing false positives due to cross-reactivity with non-target species or other food ingredients [22,23,24]. This could be achieved by the use of novel immunoreagents obtained by antipeptide antibody technology, suitable for milk species identification [25]. The characteristics, advantages and limitations of antibody-based techniques for the assessment of dairy products authenticity have been reviewed by Pizzano et al. [13]. 

ELISA has been used for species authentication in milk and dairy products since the late 1980s [13]. Hurley et al. [26] described the development of an indirect competitive ELISA, using bovine immunoglobin G (IgG) as a target, due to its high immunogenicity, to detect the presence of cow’s milk in other types of milk. The sensitivity of this technique was assayed using raw, pasteurized and previously frozen cow’s milk, concluding that high temperatures caused specific epitope modification. The detection limit in this method was 1 µg/mL of bovine IgG (0.1%), highlighting its high sensitivity without cross-reactivity with other species. Another study aiming at detecting cheese adulterations also targeting bovine IgG, but applying a sandwich ELISA, was performed by the same authors [23]. This methodology allowed further lowering the sensitivity to 0.001% of bovine milk in goat soft cheese and 0.01% of bovine milk in sheep and buffalo soft cheese. 

ELISA targeting fairly thermostable proteins, such as caseins, has been proposed as a feasible alternative to detect adulterations in heat-treated milk and dairy products. Among caseins, bovine β-caseins present a high specific antigenicity, not being affected by heat treatment and having a concentration more or less stable and independent of season, climatic and feeding conditions [27,28,29]. Therefore, different ELISA have become available in the format of commercial kits for routine surveillance tests. The performance of such kits has been evaluated in different studies showing their usefulness for qualitative purposes but exhibiting inconsistencies in quantitative determinations of cheese adulteration. In 2008, Costa et al. [30] evaluated two specific commercial ELISA kits to quantify the amount of cow’s and goat’s milk added to sheep’s milk and cheese and concluded that they were more successful in detecting the adulteration in milk than in cheeses. More recently, Zeleňáková et al. [31] tested the reliability of a commercial ELISA (RC-bovino from Zeu-Inmunotec, Spain), concluding that the quantification of cow’s milk in sheep’s cheese was not exact, possibly due to modifications in the cheese matrix that take place during the manufacturing process. The same commercial ELISA kit was also used by Stanciuc et al. [32] to qualitatively detect the presence of cow’s milk in goat’s and sheep’s cheeses for confirmation of positive results obtained with a immunochromatographic method. From 73 tested samples from Romania, 67.3% of sheep’s cheeses and 79.7% of goat’s cheeses were adulterated by the addition of cow’s milk, suggesting the need to improve the quality control in the cheese industry. Another commercial kit (Casein ELISA set, SEDIUM R&D) was used by Zeleňáková et al. [33] to detect and quantify cow’s milk caseins in sheep’s milk and cheese, obtaining a calibration curve in the range of 0.5–50% using different mixtures of heat-treated milks. When applied to cheeses, the kit did not provide any relation between the presence of caseins and the increase in the cow’s milk proportion in the mixture, either using raw or pasteurized milk, concluding its inadequacy for cheese analysis. By contrary, the use of a sandwich ELISA kit (β-Lactoglobulin ELISA Set, SEDIUM R&D) targeting bovine β-lactoglobulin to detect adulterations in sheep’s milk and cheese was able to provide a quantitative analysis within 0.2–20 mg/kg [34].

Lateral flow immunoassays (LFIA) are alternative tools very easy to handle by non-expert workers. Thus, they can be applied in-field for screening purposes and are appropriate to be used by the cheese industry to quickly check and control the genuineness of the milk used along its production chain. Recently, Galan-Malo et al. [35] developed and validated a rapid test based on LFIA able to detect down to 0.5% of cow’s milk in goat’s, sheep’s or buffalo’s milk without identifying any false-positives among over 146 negative assayed samples. 

Although most available immunochemical assays concern the authentication of sheep’s, goat’s and buffalo’s milk and/or cheeses, some studies have addressed other animal species. Pizanno et al. [25] developed an ELISA based on the use of antipeptide antibodies raised against the 1–18 sequence stretch of cow’s β-casein to successfully detect the presence of low levels (0.5%, *v/v*) of cow’s milk fraudulently blended with high-valued donkey’s milk. An indirect competitive ELISA to detect cow’s milk in yak’s milk using a specific monoclonal antibody for bovine β-casein (mAb 1-9B) was developed by Ren et al. [36]. The method allowed detecting 10 µg/mL of bovine milk in yak’s milk and was not affected by any external factors such as temperature and milk treatment.

**Table 1:** Summarized information of examples on reported electrophoretic and immunochemical methods applied to species identification in dairy products.

Method	Target Species	Target Molecule	Type of Product	Sensitivity	Reference
Native PAGE	Cow	Bovine β-lactoglobulin and α-lactalbumin	Milk mixtures	3% in caprine/bovine 5% in ovine/bovine	[11]
IEF	Cow	γ_2_-and γ_3_-caseins	Ewe’s and goat’s cheeses	- ^a^	[12]
	Cow	Bovine α_s1_-casein	Donkey’s milk	5% of cow’s milk in donkey’s milk	[25]
IEF and immunoblot analysis	Cow	Bovine γ_2_-casein	Water buffalo milk and derived mozzarella cheese	0.25% bovine milk in water buffalo mozzarella cheese	[15]
CE	Cow, sheep, goat	Casein fractions and their breakdown products	Iberico-type cheeses made from cow, sheep or goat’s milk	-	[20]
	Sheep and cow	Ovine and bovine proteins	Sheep’s/cow’s milk mixtures	5% of cow’s milk in ovine/bovine milk mixtures	[18]
	Cow	Bovine α-lactalbumin	Cow’s milk in buffalo’s milk	1% of cow’s milk (limit of quantification of 3.1%)	[19]
	Cow	α-lactalbumins and β-lactoglobulins	Goat’s and ewe’s cheeses	1% (cow’s milk)	[21]
Capillary IEF	Cow	Products of plasmin hydrolysis of bovine and water buffalo β-casein	Water buffalo’s milk	1% (cow’s milk)	[17]
ELISA	Goat	Caprine IgG	Sheep’s milk	0.5% (of goat’s milk in sheep’s milk)	[28]
Indirect Competitive ELISA	Cow	Bovine IgG	Goat’s, sheep’s and buffalo’s milk	1.0 µg/mL of bovine IgG (0.1%)	[26]
	Cow	mAb 1-9B	Yak’s milk	1% (10 µg/mL) of cow’s milk in yak’s milk	[36]
Competitive ELISA	Cow	Bovine β-casein	Donkey’s milk	0.5% of cow’s milk in donkey milk	[25]
Indirect ELISA	Cow	Bovine β-casein	Raw and heated goat’s milk	2% of cow’s milk in goat’s milk	[29]
	Cow	Bovine β-casein	Goat’s and sheep’s milk cheeses	- ^a^	[37]
Sandwich ELISA	Cow	Anti-bovine IgG antibody	Dairy products	0.001% cow´s milk in buffalo or sheep milk; 0.01% cow’s milk in goat’s milk; 0.001% in goat cheeses and 0.01% in buffalo and sheep cheeses	[23]
ELISA kits	Cow and goat	Bovine or caprine protein β-lactoglobulin	Ewe’s milk and cheese	∼0.2% of cow and goat’s milk in ewe’s milk Not adequate for quantitative measures in cheese	[30]
ELISA kits	Cow	Bovine IgG	Sheep’s milk and cheese, and commercial “Bryndza”	0.5% raw and 50% pasteurized cow milk in sheep’s milk; 0.5% raw and low pasteurized and 5% high pasteurized cow milk in sheep’s cheese	[31]
Sandwich ELISA kit	Cow	Bovine β-lactoglobulin	Sheep’s dairy products	0.2 ppm (mg/kg)	[34]
LFIA	Cow	Specific bovine immunoglobulins (IgG)	Buffalo, sheep and goat raw milks	0.5% of cows’ milk	[35]
Optical immunoassay	Cow	Bovine k-casein	Raw and pasteurized cow’s and goat’s milks	0.04% (cow’s milk in goat’s milk)	[38]
QCM immunosensor	Cow	Bovine k-casein	Cow’s and goat’s milks	1 ppm (cow’s milk in goat’s milk)	[39]

CE, capillary electrophoresis; ELISA, enzyme linked immunosorbent assay; IEF, isoelectric focusing; LFIA, lateral flow immunoassay; PAGE, polyacrylamide gel electrophoresis; QCM, quartz crystal microbalance; ^a^ not reported.

### 2.3. Chromatographic and Mass Spectrometry Techniques

**Figure 2:**
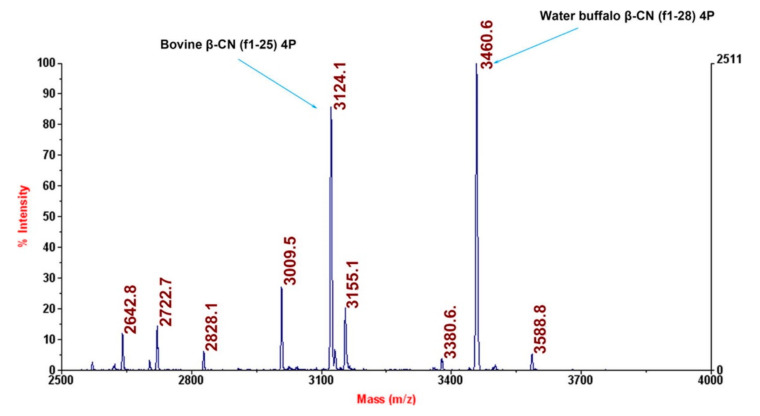
MALDI-TOF-MS-based identification of the proteotypic species WB β-CN (f1-28)4P and B β-CN (f1-25)4P as deriving from the CN fraction of WB milk containing 50% *v/v* B counterpart, which was preventively subjected to HA-based phosphoprotein enrichment and trypsinolysis. Reported is a partial view of the mass spectrum, showing well resolved (ΔM = +336 u), intense signals associated with the proteotypic species. WB β-CN (f1-28)4P (*theor*. MH^+^ = 3460.3); B β-CN (f1-25)4P (*theor*. MH^+^ = 3124.3). Reprinted from [16] with permission from Elsevier.

Up until now, different chromatographic techniques, including either gas or liquid chromatography, have been applied to authenticate dairy products because of their relative simplicity and speed, as well as possibility of automation [7,22]. High-performance liquid chromatography (HPLC) with ultraviolet (UV) detection was firstly used for the separation of the different casein fractions, relying on both normal (NP) or reverse-phase (RP) columns to identify cow’s milk in goat’s and sheep’s milk [40,41,42,43]. However, UV detection has drawbacks related to low specificity in the presence of co-eluting peaks or interferents. Thus, during the past decade, the technological advances, mainly in the area of mass spectrometry (MS) detection, have steadily replaced UV detectors, whenever the detection of food frauds is concerned. Soft-ionization techniques, such as electrospray ionization (ESI) and matrix-assisted laser desorption ionization (MALDI), have made possible to accurately analyze proteins and peptides, therefore allowing their use as reliable biomarkers for dairy product authentication. Peptides as biomarkers present advantages over proteins, which are affected by thermal processing [44]. Owing to the specificity, fastness, sensitivity and high reproducibility of the mass spectra, several methodologies based on MALDI time-of-flight mass spectrometry (TOF MS) have been developed, so far, to obtain informative fingerprints of milk proteins towards dairy product authentication [45] (Figure 2).

Based on MALDI-TOF MS analysis of intact proteins of different milk species, Cozzolino et al. [46] suggested α-lactalbumin and β-lactoglobulin as markers for detecting cow’s milk added to sheep’s and buffalo’s milk or cheese. The authors also demonstrated the usefulness of the method in detecting the addition of powdered to fresh milk based on the presence of lactosylated forms originated by heat processing. The analysis of entire proteins by direct MALDI-TOF MS coupled to unsupervised statistical analysis was also successfully proposed for milk authentication by Di Girolamo et al. [45] and Nicolaou and Goodacre [47]. Identical results were obtained by Kuckova et al. [48] regarding the identification of the species of origin in milk, though the same was not verified when the method was applied to analyze commercial cheeses, which could be attributed either to protein profile modifications or to adulteration of ovine and caprine cheeses. Recently, Rau et al. [49] demonstrated the feasibility of MALDI-TOF MS combined with a small in-house validated database, containing more than 150 reference spectra of milk and cheese, as a rapid, easy and robust method to identify the species of origin in mozzarella and white brined cheeses. The direct protein extraction without applying a tryptic digestion step allowed performing the analysis in less than 30 min with reduced analytical costs.

Other approaches have relied on a bottom-up proteomic strategy, based on MS analysis of peptides obtained after enzymatic digestion [49,50,51,52]. Calvano et al. [50] reported several bovine-specific peptide markers in milk tryptic digests that can be useful for detecting adulterations by cow’s milk addition to goat’s or sheep’s milk. Since the detection of sheep’s milk adulterated with goat’s milk is a difficult task because of their similar protein profiles, two goat-specific peptide markers assigned to κ-casein were identified [50]. Caira et al. [51] used a MALDI-TOF MS method to simultaneously determine the presence of water buffalo’s and cow’s milk in Italian water buffalo’s mozzarella cheese. Since crossbreeding with other water buffalo breeds has been avoided in indigenous Mediterranean Italian buffalo, these animals generally exhibit reduced milk protein polymorphisms when compared to other international breeds. Therefore, hundreds of milk samples (Italian and from several other countries) were analyzed, aiming at identifying signature peptides associated with water buffalo origin for the authentication of PDO products [51]. Caseins were the target proteins owing to the identified differences between indigenous and international breeds, namely the unique presence of a β-CN A variant and an internally deleted αs1-CN (f35-42) variant in international water buffalo milk samples. The peptidomic approach allowed the identification of several tryptic signature peptides as molecular marker candidates to detect the addition of imported water buffalo’s milk in Italian PDO products, as well as adulterations with cow’s milk blending. The proposed methodology enabled the specific detection of international water buffalo and bovine caseins down to 2% and 0.78%, respectively. MALDI-TOF MS has also been proposed to detect the adulteration of water buffalo’s ricotta with bovine milk based on a specific peptide marker, corresponding to the region 149–162 of β-lactoglobulin, enabling its detection down to 5% [52]. Nardiello et al. [53] proposed the use of a nano LC−ESI-ion-trap tandem mass spectrometry (nano LC-ESI-IT-MS/MS) methodology combined with a database post-processing to validate peptide sequence assignments and determine the species of origin in milk samples. Bovine species-specific peptides originated from αS1-casein and β-lactoglobulin were identified as suitable authenticity markers with detection levels as low as 1%.

MALDI-TOF MS has also been referred to as a tool for selecting the most suitable peptide makers in further analysis by liquid chromatography coupled to mass spectrometry (LC-MS) [54,55,56]. In fact, LC-MS has been increasingly applied in food analysis owing to its powerful capacity in detecting and quantifying specific analytes in complex mixtures, offering particularly enhanced selectivity and sensitivity when multiple reaction monitoring (MRM) scanning is applied [57,58]. Cuollo et al. [54] used two techniques, namely MALDI-TOF MS and LC-ESI/MS, to detect specific signature peptides to differentiate cow’s, sheep’s, goat’s and water buffalo’s milks, with both approaches providing similar sensitivities (1% for caprine and 0.5% for the other species). αs1-CN (f8-22) peptide was selected as a convenient marker for cow’s, sheep’s and water buffalo’s milk, while αs1-CN (f8-22) was for goat’s milk. MALDI-TOF MS data were tentatively used to perform quantitative analysis based on synthetically modified proteotypic peptides as internal standards, but accurate evaluation of caprine milk in quaternary mixtures was only achieved by LC-ESI-MS.

Sforza et al. [59] described an LC-MS method to evaluate the presence of cow’s milk in fresh sheep’s milk cheese targeting short marker peptides, namely αs1-CN (f1-23) and αs1-CN (f1-14), generated from proteolytic activities of the rennet enzyme chymosin and starter lactic acid bacteria, respectively. While the first peptide was degraded over time, thus being undetectable after long ageing periods, the second was frequently observed in cow’s milk cheeses. Despite this occurrence, the authors referred to its detection in hard cheeses aged for more than 30 months. Moreover, the degradation of αs1-CN (f1-23) peptide also led to other fragments that could be detected. The method allowed the detection of cows’ milk down to 1% in all the analyzed cheeses, demonstrating the usefulness of these two candidate biomarkers to assess the addition of cow’s milk in fresh sheep’s cheese [59]. Czerwenka et al. [60] developed an LC-MS method to detect the adulteration of cow’s milk in water buffalo’s milk and mozzarella cheese, targeting the whey β-lactoglobulin as an adulteration marker. Since this water-soluble protein is mainly present in the whey fraction and not in the cheese, the analyzed parts were the brine in which this type of cheese is usually sold, or in the exudate obtained after cheese centrifugation. The authors showed that sufficient amounts of β-lactoglobulin were present either in the brine or exudate, allowing the detection of adulterations with cow’s milk. The application of this method to assess 18 commercial samples of water buffalo mozzarella cheese allowed detecting three adulterated products. However, quantitative determination presented several pitfalls because of the variability the target the analyte between and within the two blended milks and the lack of an internal standard. Quantification of the fraudulent addition of bovine milk in the production of buffalo mozzarella PDO cheese was claimed by Russo et al. [57], based on UPLC-MS/MS exploiting the MRM mode, though the protein level in the studied cheeses was not taken into consideration. The use of MRM, as described in this study, allowed a highly selective and sensitive detection and quantification of the chosen proteotypic marker, even in complex matrices, by simultaneously monitoring both their parent and one or more product ions. The selection of the species-specific proteotypic marker—phosphorylated β-CN (f33-48) tryptic peptide—was performed by an untargeted LC-MS/MS analysis by means of a quadrupole TOF MS equipped with an ESI source (ESI-Q-q-TOF). Additionally, to select the best conditions for trypsin digestion, a preliminary study was conducted by MALDI-TOF MS. Overall, the method allowed targeting the marker peptides with high specificity, thus being adequate for the authentication of complex matrices such as dairy products [57]. 

Despite the claimed advantage of quantitative analysis by LC-MS methods, it must be referred that it mainly gives an estimation of the fraud extent since the protein content of milk is known to vary with different factors, with the breed and season being of most relevance [55,56,57,58,59,60,61]. Trying to overcome this aspect, Gunning et al. [58] proposed the use of MRM MS-targeting αS1-casein to detect the addition of cow’s milk to buffalo mozzarella cheese. The relative amounts of each species in binary mixtures were determined based on corresponding peptides arising from a corresponding protein strategy and the ratios of transition peak areas. Moreover, identical peptides with the same sequences in both species were used to establish the relative levels of both species of αS1-casein in the component mixtures. The method was applied in a survey of 28 products sold in UK retail and restaurants, enabling us to verify that almost 2/3 were suspicious of being adulterated with cow’s milk. An UHPLC-MS/MS method also exploiting MRM mode, using at least two transitions for each compound, has recently been reported by Ke et al. [62] to quantify cow’s whey and whole milk powder in goat’s and sheep’s milk products, including infant formula. This method allowed the simultaneous quantification of four caseins (β-CN, αs1-CN, αs2-CN, and κ-CN) and two whey proteins (α-lactalbumin, β-lactoglobulin) based on the detection of their signature peptides. Isotopic labeled signature peptides were used as internal standards to compensate the matrix effect. The method was successfully validated regarding several parameters. Calibration curves for the tryptic signature peptides presented good linearity, the limits of quantification were between 0.01–0.05 g/100 g for the target proteins and the method showed high precision, reproducibility and recovery rates. The analysis of 11 commercial samples of goat infant formula milk powder revealed some adulterations among the evaluated products [62].

Although proteomic approaches developed so far mostly rely on the target identification of marker peptides, recently an untargeted UHPLC−MS/MS high resolution MS (HRMS) combined with chemometrics, was proposed to discriminate among cow’s, goat’s and buffalo’s milk samples [63]. The approach allowed the identification of different marker compounds, suggesting β-carotene and ergocalciferol for cow’s and water buffalo’s milk identification, respectively. Moreover, the levels of octanoic, nonanoic and decanoic acids were found to be higher in goat’s than in cow’s and buffalo’s milk [63].

Recently, the development of ambient ionization techniques, such as direct analysis in real time (DART), enabled a high-throughput and easy analysis of food. The potential of this ionization technique coupled to HRMS and chemometrics was exploited for dairy product authentication, including the discrimination of cow’s, goat’s and sheep’s milk. Results showed that DART-HRMS analysis of the non-polar fraction of milk had a limited discrimination potential, probably due to the high variability in triacylglycerols (TAG) among each group of samples [64]. 

Although the application of both chromatographic and mass spectrometry techniques to dairy product authentication mainly relies on protein analysis, other compounds such as fatty acids and TAG have also been addressed for this purpose [65,66,67]. Bratu et al. [68] used GC-MS analysis of fatty acid methyl esters coupled to principal component analysis (PCA) to differentiate 25 different cheeses (including cow, goat and sheep). Although sample discrimination in 3 groups was achieved using 12 components, more studies should be performed comprising a higher number of samples, also including model cheeses made with mixtures of milk besides pure milk cheeses. Vieitez et al. [69] showed that the addition of cow’s milk to pure goat’s milk influences the TAG profile by determining the partition number (PN), which characterizes the molecular structure of TAG. The analysis of blends containing 10, 20 and 50% of cow’s milk showed that the addition of cow’s milk to goat’s milk affects the TAG profile by decreasing TAG with PN between 38 and 42, while increasing it with PN between 46 and 50. Of the 15 commercial samples evaluated, 3 presented a different TAG profile, suggesting their possible adulteration with cow’s milk. However, since there are many factors that can influence the TAG profile (breed, feeding regime, season, etc.) the study should be extended in order to further include a higher number of samples.

The summarized information about different chromatographic and mass spectrometry methods applied to species authentication in dairy products is presented in Table 2.

## 3. Spectroscopic Methods

In the last decade, different stakeholders have evidenced the need for less expensive, rapid and efficient methods for the detection of adulterations in dairy products. Accordingly, several spectroscopic methods have been developed and applied to dairy product authentication, including near infrared (NIR), mid infrared (MIR), front face fluorescence spectroscopy (FFFS), Fourier-transform infrared (FT-IR) and nuclear magnetic resonance (NMR) [6,70]. Spectroscopic techniques, in general, are considered as auspicious tools to detect adulterants in dairy products [6]. Compared with the reference methods, spectroscopic techniques present several advantages, such as fastness, simplicity, and a non-destructive nature, requiring few or no chemicals, making them suited for routine applications. However, these methods frequently require expensive equipment, extensive sample databases and chemometrics [6,7,71,72,73].

**Table 2:** Summarized information of reported chromatographic and mass spectrometry techniques applied to species identification in dairy products.

Method	Target Species	Target Molecule	Type of Product	Sensitivity	Reference
HPLC-DAD	Sheep, goat and cow	Albumines (β-lactoglobulin, α-lactoalbumin and serum albumine), globulins (immunoglobulin: IgG, IgA and IgM), proteoso-peptones and lactoferrin	Milk and cheeses	3.92% (sheep’s milk in cheese) 2.81% (goat’s milk in cheese) 1.47% (cow’s milk in cheese)	[42]
	Buffalo and cow	β-lactoglobulin	Creams	1% (cow’s milk in buffalo’s cream)	[43]
MALDI-TOF MS	Cow, buffalo, sheep, she-donkey and goat	Intact proteins	She-donkey’s and goat’s milk	0.5% (cow’s milk in She-donkey’s and goat’s milk)	[45]
	Goat, sheep and cow	Caseins and proteose peptone	Milk	2% (cow’s milk in goat’s and sheep’s milk)	[47]
	Water buffalo and cow	Four signature unphosphorylated peptides derived from β-CN A, i.e., (f49-68) Asn^68^, (f1-28) Ser^10^, (f1-29) Ser^10^ and (f33-48) Thr^41^ and two from α_s1_-CN (f35-42), i.e., (f23-34) Met^31^ and (f43-58) Val^44^	Mozzarella cheeses	0.78% (cow’s milk in PDO water buffalo’s cheeses)	[51]
	Cow and buffalo	Region 149–162 of bovine β-lactoglobulin	Water buffalo’s ricotta PDO cheese	5% (cow’s milk in buffalo’s cheese)	[52]
	Goat	αs1-CN f8-22 and αs1-CN f4-22	Milk mixtures	0.5% (goat’s milk in milk mixtures)	[54]
	Sheep, goat, buffalo and cow	γ2-caseins and γ3-caseins in the four species; α-lactalbumins in bovine, buffalo and goat milk; β- CN fragments (98–207) in goat and ovine milk; β-lactoglobulin in goat milk, proteoso peptones p.p.8.I., in bovine milk and β-casein fragments (1–68) and (69–209) in buffalo milk	Fresh raw cow’s, buffalo’s, sheep’s and goat’s milk	5% (cow’s milk in goat’s milk)	[74]
	Goat, sheep and cow	Intact phospholipids	Milk	- ^a^	[75]
LC-MS	Sheep and cow	Fragments 1–14 and 1–23 from α_S1_ casein	Fresh sheep’s milk cheeses	1% (cow’s milk in sheep’s cheese)	[59]
LC-MS/MS	Cow, buffalo, sheep and goat	β-lactoglobulin variants A and or α-lactalbumin	Buffalo’s, sheep’s and goat’s Italian ricotta cheese	0.5% (cow’s whey in ricotta cheeses from the other species)	[55]
LC-ESI-MS	Goat	α1-CN f4-22 variant A and B	Milk mixtures	- ^a^	[54]
LC-ESI-MS/MS	-	Caseinomacropeptide (CMP) and pseudo-CMP	Milk	1 µg/mL (CMP and pseudo-CMP in milk)	[76]
	Cow, buffalo, sheep and goat	Species-Specific Peptides: Goat (YLGYLEQLLK), sheep (TPEVDNEALEK), buffalo (AFKPTELGEVITK) and cow (AMKPWIQPK)	Milk and cheeses	- ^a^	[77]
HPLC-ESI-MS, MALDI-TOF MS and MS/MS	Goat	Variant D of caprine β-casein	Italian goat’s milk	- ^a^	[56]
UPLC-ESI-MS/MS	Cow and buffalo	β-casein f33-48 transitions	PDO buffalo’s mozzarella	0.001% (cow’s milk in buffalo’s cheese)	[57]
UHPLC-MS/MS	Goat, sheep and cow	Caseins (β-casein, α_s1_-casein, α_s2_-casein, and κ-casein) and major whey proteins (β-lactoglobulin and α lactalbumin)	Cow’s milk whey, whole milk powder and goat’s milk infant formula	0.01–0.05 g/100 g (cow’s whey and whole milk powder in goat’s or sheep’s milk products including infant formula)	[62]
UHPLC-MS/MS	Cow	Peptide LRPVAAEIYGTK, VDSALYLGSR (corresponding to amino acid residues 93–104 and 333–342 of bovine lactoferrin, respectively)	Dairy products, include infant formula and whey proteins	0.3 mg/100 mg (cow’s lactoferrin in infant formulas)	[78]

ESI, electrospray ionization; HPLC, high performance liquid chromatography; MALDI-TOF, matrix-assisted laser desorption ionization time-of-flight; MS, mass spectrometry; UHPLC, ultra-high performance liquid chromatography; ^a^ not reported.

**Figure 3:**
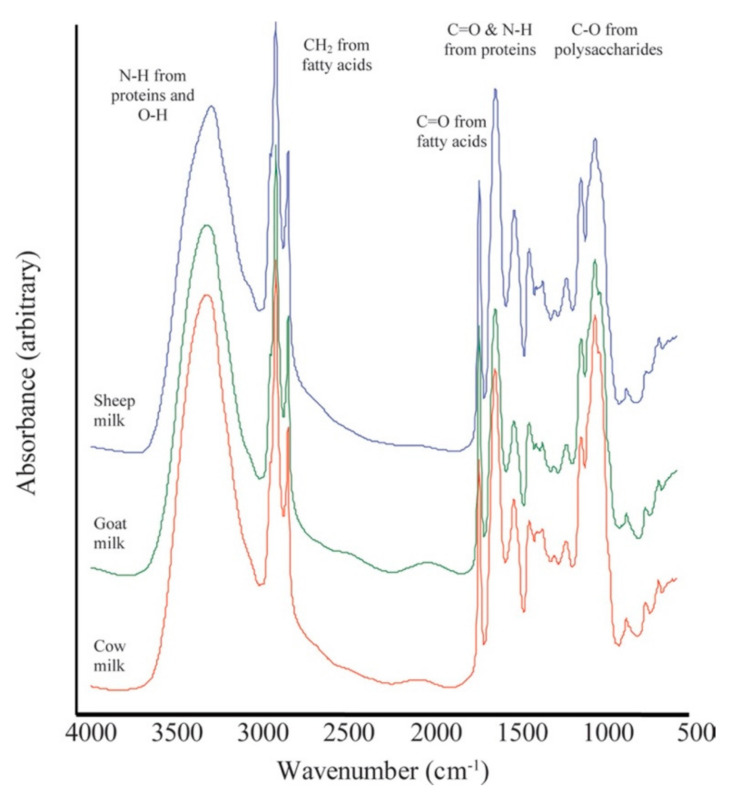
Fourier transform infrared spectra for pure cow, goat, and sheep milk. These spectra are offset to allow visualization of any difference. Reprinted from [72] with permission from Elsevier.

Techniques relying on infrared (IR) radiation have the advantage of allowing the analysis of samples, either in the solid or liquid state, which can provide specific spectra using selected frequency ranges [79]. Infrared spectroscopy is based on the measurement of the fundamental vibrations of molecules, with the collective effect from each functional group that has a specific vibrational frequency, resulting in a unique molecular fingerprint. Both mid-infrared (MIR, approximately from 400–4000 cm^−1^) and near-infrared (NIR; approximately from 4000–14,000 cm^−1^) have been applied to authenticate dairy products. FT-IR, considered as a fast biochemical fingerprinting technique, has already been described in the analysis of cheese quality, quality control of milk and cheese ripening process, as well as authenticity assessment [80]. FT-IR was proposed by Nicolaou et al. [72] to detect and quantify the percentage of cow’s milk adulteration in mixtures of different types of milk, namely goat, sheep and cow, suggesting its potential applicability in the food industry. From a qualitative point of view, the spectra of cow’s and goat’s milk were very similar but showed quantitative differences that were mainly evidenced in sheep’s milk (Figure 3). FT-IR also allowed the discrimination of cow’s, sheep’s and water buffalo’s milks and their classification by hierarchical clustering and PCA on the basis of Euclidean distance and Ward’s algorithm [81]. Recently, FT-IR was employed to verify the species of origin of Halloumi cheese, a traditional Cypriot cheese that should be made either with goat’s or sheep’s milk. The interpretation of the obtained spectra was carried out by chemometric analysis using SIMCA software, enabling the differentiation of cow’s milk or goat/sheep’s milk products, with supervised orthogonal partial least squares discriminant analysis [82]. Unsupervised and supervised methods applied to FT-IR spectra to assess goat’s cheese and yogurt adulterated by cow’s milk addition at the levels of 10%, 15% and 20% were evaluated by Teixeira et al. [83]. Both approaches showed good results as they were able to distinguish the adulterated products. Moreover, the use of an interval partial least-square (iPLS) algorithm allowed the researchers to dramatically reduce the number of variables which, according to the authors, may represent a step towards the development of cheaper portable devices.

Brandao et al. [84] developed a front-face and time-resolved fluorescence method for a rapid screening of frauds in goat’s milk powder by the addition of cow’s milk powder. Compared with steady-state spectroscopy, time resolved fluorescence offers some advantages because it measures the time dependence (lifetimes, which are determined by fluorescence intensity decay) of the fluorescence instead of its emission intensity. Additionally, fluorescence lifetime is not altered by photo bleaching, it is independent from the fluorescence intensity and largely independent of fluorophore concentrations. The intensity levels of excitation and emission were measured at 315 nm and 468 nm, respectively, whose results showed increased intensity in samples related with increasing addition of cow’s milk powder. This study successfully demonstrated fluorescence lifetimes as a promising technique for the application in real-time assessment of frauds in goat’s milk powders, providing a potential tool for food authentication, particularly dairy products.

Synchronous fluorescence spectroscopy is another technique, generally combined with multivariate analysis, applied to detect adulterations in food. Velioglu et al. [85] exploited this technique to detect the addition of milk in buffalo’s milk and to discriminate both species. The developed method showed a limit of detection of 6% of cow’s milk and a good distinction between the spectra of both species. These differences were found in the range of 400–550 nm, with breaks of 10 nm, which were further analyzed using PCA to distinguish the two species and by partial least square (PLS) analysis to estimate the level of cow’s milk adulteration in buffalo’s milk samples [85]. This technique has also proved its usefulness in discriminating cow’s, goat’s, ewe’s and buffalo’s milk and estimate the level of cow’s milk addition in the case of samples classified as being binary mixtures [86].

## 4. DNA-Based Methods

DNA-based methods relying on polymerase chain reaction (PCR) have been widely applied to detect adulterations in foods from both plant [87] and animal [88,89,90] origins, including dairy products [8,91] because of their simplicity, high sensitivity and high specificity. They benefit from the high thermal stability of DNA molecules, which is particularly relevant when analysing processed foods, and are independent from immunochemical recognition, making them not susceptible to cross-reactivity. The ubiquity of nucleic acids in every type of cell and particularity in healthy mammary glands, which have high numbers of leucocytes and epithelial cells that are transferred to the milk, is another advantage to highlight [4]. During cheese making, these cells are concentrated and allow the isolation of DNA to discriminate the species.

For the successful application of PCR-based methods, the extraction and isolation of DNA is a crucial task. In food matrices, the presence of hydrolytic enzymes may affect the DNA integrity and, consequently, its amplification [7]. A recent review details different aspects related to DNA extraction from dairy products as well as factors including processing, transport and handling, which may influence the applicability of DNA-based methods for the authentication of these products [8]. 

Several PCR-based methods have been widely applied to species identification in dairy products, namely PCR-RFLP (restriction fragment length polymorphisms), species-specific PCR, multiplex PCR and real-time PCR. Most of these methods rely on the amplification of mitochondrial genes because of their high number in animal cells, thus increasing the sensitivity of the assays. More recently, other DNA approaches such as high-resolution melting (HRM) analysis, droplet digital PCR (ddPCR), loop-mediated isothermal amplification (LAMP), next-generation sequencing (NGS) and biosensors have provided innovative alternatives for species authentication in dairy products. Table 3 presents the summarized information of reported methodologies based on DNA analysis for species authentication in dairy products.

### 4.1. PCR-RFLP

PCR followed by RFLP analysis relies on the amplification of a selected marker followed by digestion with restriction enzymes that recognize specific loci, providing species-specific fragment patterns. This technique has been long applied to food authentication, including dairy species identification due to its simplicity, low-cost and aptitude for routine analysis [88,89]. Plath et al. [92] reported the first PCR-RFLP method, targeting the β-casein gene and combined with polyacrylamide gel electrophoresis to identify bovine milk in ovine or caprine milk and cheeses. Since then, other PCR-RFLP methods coupled to agarose gel electrophoresis were further proposed to identify milk species in dairy products, targeting mostly casein [95,98] and *cytb* genes [93,94]. PCR-RFLP methods applied to dairy products provide mainly species differentiation, namely cow, sheep, goat and buffalo, although some methods allow achieving levels of detection [92,95].

### 4.2. Species-Specific PCR

Species-specific PCR is a standard technique that has been successfully applied to the species authentication of complex and processed foods, including dairy products, owing to its simplicity, high specificity and high sensitivity [8,88,89,91]. It relies on the accurate design of primers to allow the amplification of a species-specific sequence based on end-point PCR. Different works have proposed the use of species-specific PCR followed by agarose gel electrophoresis for detecting milk species in dairy products, mainly cow, goat and sheep, but also other less commonly used such as buffalo, camel, mare and yak (Table 3). The methods have been successfully applied to authenticate processed dairy products, namely pasteurized milk, freeze-dried milk, powder milk, UHT milk, fresh and aged cheeses, cream, yogurt and butter (Table 3). Most works have relied on the amplification of mitochondrial DNA, with the 12S rRNA gene being the most frequent target, followed by the 16S rRNA, *cytb* and D-Loop regions. Generally, species-specific PCR methods allow reaching low sensitivity, down to levels in the range of 0.1–1%.

The use of two or more pairs of primers in the same reaction can allow the simultaneous detection of multiple species based on multiplex PCR. The development of duplex or multiplex PCR approaches has also been attempted for the simultaneous detection of different species in dairy products, resulting in faster and lower-cost authentication tools. Bottero et al. [105] developed a multiplex PCR method that was able to simultaneously identify cow, sheep and goat targeting the mitochondrial 12S rRNA and 16S rRNA genes, achieving a sensitivity of 0.5% of cow’s milk in goat’s milk. Following this work, Mafra et al. [106] developed a duplex PCR method based of the measurement of band intensity of agarose gel electrophoresis that allowed detecting 0.1% of bovine milk in sheep’s cheese and quantifying adulterations with bovine milk within 1–50%. Subsequently, the same authors developed a duplex PCR with similar sensitivity and quantification range of cow’s milk in goat’s cheese [107]. Both approaches were successfully validated with blind cheeses and applied to commercial pure and mixture cheeses. Multiplex PCR assays have also been combined with capillary electrophoresis, as described by Gonçalves et al. [110], who were able to simultaneous detect cow, sheep, goat, and water buffalo in dairy products. The applications of multiplex PCR to dairy product authentication are summarized in Table 3.

### 4.3. Real-Time PCR

**Figure 4:**
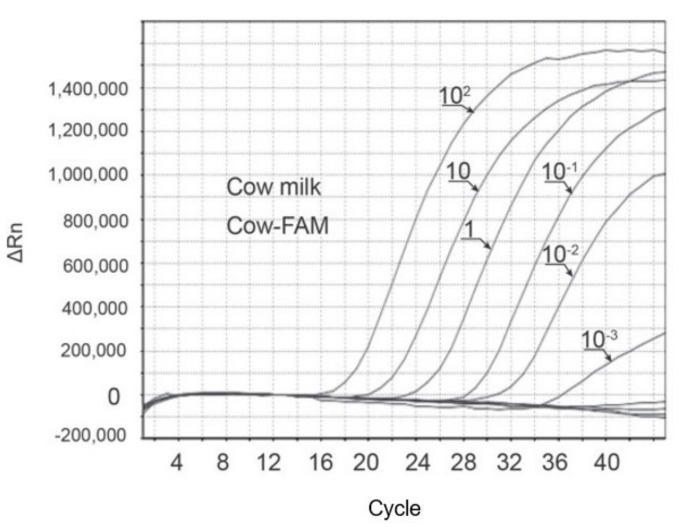
Real-time PCR amplification curves targeting the 12S rRNA gene of cow with a TaqMan probe using serially diluted DNA (ng) extracted from cow’s milk. FAM, fluorescent reporter 6-carboxyfluorescein, ΔRn, change in normalized reported value. Reprinted from [132] with permission from Elsevier.

Real-time PCR is based on monitoring the amplified target fragments along the amplification cycles with the use of fluorescent reported molecules. It provides several advantages over end-point PCR, namely higher sensitivity, specificity and reproducibility, as well as a low level of cross-contamination and reduced time of analysis. The capacity of quantifying the starting amount of a specific DNA target, which is intrinsic to its ability of measuring the target product at early stages of amplification (exponential), is a key advantage of real-time PCR [145]. Therefore, real-time PCR has been the technique of choice in many control and diagnostic laboratories for food analysis aiming at food authentication, GMO quantification and allergen analysis [88,89,90,146]. The use of DNA binding dyes, such as SYBR Green I, to monitor the real-time PCR amplification is the simplest and most economic approach, but it requires a melting curve analysis as a post-PCR verification of specificity. The hydrolysis fluorescent probes, such as the TaqMan™, designed to bind to a specific region of the target DNA have been preferred owing to the increased method specificity, but also to their relatively simple design and multiplexing capacity, without requiring melting curve analysis [145]. As a result, most real-time PCR methods applied to dairy product authentication have used TaqMan probes (Table 3). Like for end-point PCR assays, real-time PCR assays have targeted mostly sequences of the mitochondrial 12S rRNA gene, followed by the *cytb* gene. The lowest relative sensitivities achieved with real-time PCR were similar to end-point PCR (0.1% for cow’s milk in dairy products), though a much lower absolute detection was attained (down to 1–5 pg of milk DNA) (Figure 4) (Table 3).

The use of multiple specific primer and probe sets targeting more than one species simultaneously has been particularly exploited in dairy product authentication. The first multiplex approach was proposed by Cottenet et al. [130] to simultaneously detect cow’s and buffalo’s milks using specific fluorescent probes targeting the *cytb* gene of both species. Rentsch et al. [131] developed two multiplex real-time PCR systems with TaqMan probes to simultaneously detect the main milk species targeting mitochondrial and nuclear genes, which were designated as Allmilk and Allcheese, respectively. Both systems were applied in the estimation of cow’s milk of fresh and ripened model cheeses, with the nuclear systems revealing the highest specificity and quantitative performance. Later on, the same group of researchers developed three triplex real-time PCR methods with TaqMan probes targeting the 12S rRNA gene to simultaneously detect an endogenous control sequence and two species, namely cow and mare [132], cow and goat [133], sheep and goat [134] and camel and cow [135]. The approaches were successfully applied to processed dairy products, achieving high sensitivities down to few pictograms of DNA (Table 3).

### 4.4. HRM Analysis

High-resolution melting (HRM) analysis is a post-PCR approach based on monitoring the gradual denaturation of double-stranded DNA of amplified fragments, allowing us to detect small nucleotide differences. It enables performing genotyping, gene mapping, allelic and single nucleotide variant discrimination, and barcode analysis. As a result, HRM has proven to be a rapid, simple and cost-effective tool, providing wide applicability in several research and diagnostic areas, with particular emphasis for species differentiation from diverse food origins [87,90,147,148,149]. HRM analysis targeting the mitochondrial D-loop region was able to discriminate bovine, ovine and caprine species in cheeses. Moreover, it allowed detecting cow’s milk down to 0.1% and estimating the ratio of goat to sheep milk [136]. The same group of researchers developed a duplex HRM method targeting the 12S rRNA gene to differentiate cow’s and buffalo’s milks, which allowed detecting cow’s milk in Mozzarella cheese down to 1% and also estimating the ratio of bovine to buffalo milk [137].

### 4.5. ddPCR

Droplet digital PCR (ddPCR) is a breakthrough technology based on partitioning individual amplifications into separate compartments using droplets or chambers, providing accurate quantification of target DNA. ddPCR enables ultrasensitive and absolute DNA quantification without the need of a standard curve, which is an advantage over real-time PCR. It has been applied to clinical diagnostics, pathogen detection and food analysis, particularly gene-edited plants, GMO detection and authentication of meat products [150,151,152]. Recently, a ddPCR method targeting the *cytb* gene was developed to detect cow’s and buffalo’s milk in mozzarella cheese [138]. The method provided a sensitivity down to 0.1% of cow’s milk in cheese, which was identical to real-time PCR, but higher than end-point PCR, IEF and HPLC-UV (0.5–1%). The authors concluded that, despite the need for qualified personnel, the costs of ddPCR are comparable to those of the official IFE method and real-time PCR, considering it as an effective tool to detect adulterations at trace levels [138].

### 4.6. LAMP

Loop-mediated isothermal amplification (LAMP) is a technique that relies on the design of a set of primers that allow specific, sensitive and rapid detection of a DNA target under isothermal conditions. LAMP enables visual monitoring, providing simple, cost-effective and field applications. It is the most widely used isothermal amplification technique, being applied to food safety evaluation regarding foodborne pathogens, food allergens, GMO detection and botanical/animal species authentication [87,153,154]. LAMP has also been applied for species identification in dairy products [139,140] (Table 3). A LAMP method was developed to specifically target the D-loop region and visually detect up to 5% of cow’s milk/meat in mixtures with buffalo counterparts [139]. Kim and Kim [140] proposed a duplex LAMP method for the on-site detection of cow’s and goat’s milk using a portable fluorescence device. The method achieved a sensitivity of 0.1 and 1 pg of cow’s and goat’s DNA, respectively, and 2% for both species in milk mixtures.

### 4.7. NGS

Next-generation sequencing (NGS) technologies have revolutionised the mode of analysing DNA by providing high-speed sequencing and multiple/parallel reads, with a resultant marked reduction in cost per base. It is becoming a standard approach in many research areas, including applications to food analysis, such as foodborne microorganism detection and food authentication [87,90,155,156]. Despite the high potential of NGS for food authentication, its application to dairy foods is still limited. NGS with ion torrent technology targeting three regions of two mitochondrial genes enabled the identification of milk species in dairy products, namely goat, sheep, cow and buffalo [141]. Additionally, NGS identified different dairy species mitotypes and the presence of human DNA as a possible marker to verify the level of hygiene of dairy products.

### 4.8. Fingerprint Techniques

In addition to the demonstrated feasibility of DNA-based methods for species authentication in dairy products, they have also been challenged to identify particular breeds associated with premium dairy products. For this purpose, non-target fingerprint techniques, such as randomly amplified polymorphic DNA (RAPD), have been exploited. RAPD is a simple and economical technique that uses a single arbitrary primer to generate band fingerprint profiles. After assaying several RAPD primers, Cunha et al. [157] identified two of them capable of differentiating milks of adulterant breeds of Serra da Estrela sheep breeds used to produce PDO cheeses. To overcome the problems of low reproducibility associated with RAPD and to be able to detect adulterant breeds in PDO cheeses, the authors identified discriminatory bands that, based on their sequence, were designated as sequenced characterized amplified region markers (SCAR). The design of new SCAR primers to amplify small fragments allowed the development of a PCR-SCAR method that could be effectively applied to identify a common milk adulterant breed of Serra da Estrela PDO cheese.

Microsatellites or simple sequence repeats (SSR) are fingerprint DNA markers that rely on PCR amplification with a set of primers to target tandem repeated motifs of 2–6 bp flanked by highly conserved sequences. The different numbers of repeats in the microsatellite region are the identified polymorphisms. The high polymorphic degree and reproducibility of SSR markers allow species identification, but mostly breed/variety or even individual identification, thus being particularly useful in food traceability studies [158]. Sardina et al. [159] described the use of SSR markers to discriminate among the most important Sicilian dairy goat breeds, aiming at the authentication of Girgentana dairy products. The authors identified three specific SSR markers that could be applied as a genetic traceability system of Girgentana dairy products, allowing the detection of adulterations due to Maltese and Derivata di Siria goat’s milk breeds.

## 5. (Bio)Sensors

Sensors are devices able to measure a physical quantity and convert it into a signal that can be read by an instrument. Chemical sensors measure chemical substances by chemical or physical responses, which can be designated as biosensors when using a biorecognition element [160]. The electronic tongue is an array device of non-specific and low-selective chemical sensors, possessing high stability and cross-sensitivity to different compounds. Dias et al. [161] developed a potentiometric electronic tongue with 36 cross-sensitivity sensors that was able to differentiate the five basic tastes (salty, sweet, acid, bitter and umami) and further detecting adulterations of goat’s milk with cow’s milk. Cross-validation of a model based on linear discriminant analysis of the recorded signal profiles allowed discriminating goat, cow and goat/cow raw skimmed milks with satisfactory sensitivity and specificity (over than 87% and 70%, respectively), suggesting its capacity in distinguishing the different species in various milk samples [161].

Biosensors base their principle on the direct recognition of a biological interaction between a receptor and the target molecule (proteins or DNA, immuno-or genosensors, respectively) by a transducer that produces a measurable signal. They can provide simple, fast, high-throughput, multitarget and low-cost detection, being considered as emerging and attractive tools for food analysis, with applications on GMO detection [154], food authentication [89] and allergen analysis [162]. Regarding dairy foods, recent studies have proposed both immunosensors [38,39] (Table 1) and genosensors [142,144] (Table 3) for species authentication. A miniaturized immunosensor with optical transduction based on ten planar silicon nitride waveguide Broad-Band Mach–Zehnder interferometers, targeting bovine k-casein, was developed by Angelopoulou et al. [38]. The approach provided the determination of cow’s milk in goat’s milk based on a competitive immunoassay, achieving a sensitivity of 0.04% (*v/v*) and a dynamic range of 0.1–1.0% (*v/v*) of cow’s milk in goat’s milk. The analytical performance of the proposed immunosensor was favorably compared with a competitive ELISA developed using the same monoclonal antibodies, but in a much shorter period of time (10 min) than ELISA (2 h). The immunoassay was considered a fast and sensitive tool, being suitable for incorporation into portable devices, thus having high potential for on-field applications [38]. Sakti et al. [39] developed an immunosensor with piezoelectric transduction (quartz crystal microbalance) for the detection of cow’s milk as an adulterant of goat’s milk. The method used a specific polyclonal antibody targeting a protein of 208 kDa (k-casein) as a marker of cow’ milk, not identified in goat’s milk, achieving a sensitivity of 1 ppm of cow’s milk.

Beltramo et al. [142] carried out a validation process for the low-cost and -density (LCD) array (MEAT 5.0 version) kit for food forensics based on a DNA biochip technology (microarray) that simultaneously detects 24 animal species, based on the analysis of PCR fragments (115–125 bp) of the 16S rRNA gene with specific capture probes. The LCD array kit was successfully validated to analyze mixtures of meats or milks, achieving limits of detection of 0.5% or 0.1%, respectively. Moreover, the assay did not show differences in the performance after analyzing heat treated mixtures, exhibiting high robustness regarding several key parameters and food ingredients.

Kounelli and Kalogianni [143] developed a DNA-based method that relied on hybridization of species-specific oligonucleotide on the surface of fluorescent microspheres, followed by flow cytometry analysis. The method consisted of DNA amplification with species-specific labeled primers targeting the *cytb* gene of each species (cow, sheep and goat), followed by hybridization of the single-strand biotinylated PCR products with species-specific oligonucleotide probes, carrying a NH_2_ group at the 5′-end, which were attached to the surface of three different sets of carboxylated microspheres. The obtained hybrids were detected via a streptavidin–phycoerythrin conjugate, whose fluorescent signal is proportional to the DNA amount and achieved a sensitivity down to 0.01% of cow’s milk in goat’s milk and 0.05% in sheep’s milk. The method was successfully applied to detect milk species in milk mixtures and yogurts, exhibiting high reproducibility [143].

Recently, the same research group [84] developed a paper-based DNA biosensor for the detection of cow’s, sheep’s and goat’s in dairy products. Similarly to the above described approach, the method consisted of a first step of DNA amplification with biotin-labeled species-specific primers. Then, the single-strand biotinylated PCR products were hybridized with species-specific DNA probes carrying a poly-dA tail at one end and applied on the conjugate pad of the biosensor together with streptavidin-functionalized gold nanoparticles that provided the observation of the results by the naked eye. The biosensor revealed high specificity and high absolute (1.6 fmol of cow’s and goat’s and 3.1 fmol of sheep´s PCR products) and relative (0.01% of adulterant in yogurt) sensitivity, as well as good reproducibility.

## 6. Conclusions and Future Prospects

Species identification in milk and dairy products has been the subject of an increasing number of reports because of its importance regarding food authentication, but also in response to the growing consumers’ demands for label transparency. So far, several methodologies have been proposed to determinate the species authentication in dairy products, relying on both proteins and DNA markers. Other techniques based on spectroscopy are also increasingly considered in the determination of food authenticity due to advantages related to sample preparation, rapidity, non-destructiveness, easy performance and potential for on-field use, although the need for expensive equipment, adequate databases and multicomponent analysis might restrain their use. The resumed advantages and drawbacks of the main techniques used for species authentication in dairy products are presented on Table 4. This information can be critically useful for selecting the method(s) for species authentication in dairy products, according to the intended application. One issue that should be specifically considered regards food processing since, depending on the selected analytical method, it might lead to false negative results as in the case of the immunochemical assays, or decreased sensitivity in the case of DNA-based methods and other techniques.

Of the protein-based methods, the proteomic approaches using MALDI-TOF MS have revealed a high number of advances in species identification in dairy products, particularly when combined with unsupervised statistical analysis. With the availability of databases with the reference spectra of milk and cheese proteins, the development of rapid and robust methods that do not require prior protein extraction and digestion is expected. It is also important to refer to the effectiveness of MALDI-TOF MS for selecting the suitable marker peptides for further bottom-up proteomic strategies, particularly by liquid chromatography coupled to tandem mass spectrometry. Despite the great technological advances in MS instrumentation and methods, the costly equipment and the need for specialized personal and databases are drawbacks that disable their wide application for routine analysis.

DNA-based methods have played an important role in species authentication of dairy products owing to their high specificity and sensitivity, simple performance and low/medium cost of analysis. Particularly, real-time PCR with specific probes targeting mtDNA markers has provided a high number of methods, with the advantages of multiplexing and quantitative analysis. More recently, ddPCR has provided promising alternative methods to real-time PCR, with the advantage of not requiring calibration curves for quantitative analysis, thus more advances in their application to authenticate dairy products being expected in the near future. The advent of high-throughput sequencing technologies has also shown applicability to dairy product authentication with the main advantage over Sanger sequencing of enabling multiple species identification in complex mixtures.

Biosensors are considered cutting-edge approaches for high-throughput, simple, fast and low-cost detection, with aptitude for multiplexing and on-site analysis. Despite their advantages, applicability to dairy species authentication, both as immuno- or genosensors, is still limited, being expected to increase in the near future. The combination of LAMP and biosensor is prospected to provide highly specific, sensitive and on-site analysis. However, in biosensing analysis, considerable efforts are still required to provide quantitative analysis and applicability to processed foods. 

## Figures and Tables

**Table 3:** Summarized information of reported DNA-based methods applied to species identification in dairy products.

Technique	Target Species	Application	Target Gene	Sensitivity	Reference
PCR-RFLP	Cow, sheep and goat	Milk and cheese	β-casein	0.5% (cow’s milk in goat’s and sheep’s milk)	[92]
	Cow, sheep, goat and buffalo	Meat and milk	cytb	- ^a^	[93]
	Buffalo, cow and sheep	Milk	SSR marker and *cytb*	- ^a^	[94]
	Cow and buffalo	Mozzarella cheeses	α-, β-and κ-casein	1% (cow’s milk in buffalo’s milk mozzarella cheese)	[95]
	Cow and buffalo	Milk and butter	cytb	5% (cow’s milk in buffalo’s milk and butter)	[96]
	Cow and buffalo	Raw milk	cytb	- ^a^	[97]
	Cow, goat, and sheep	Raw and powder milks, pasteurized cream, and hard and semi-hard cheeses.	κ-casein	- ^a^	[98]
Species-specific PCR	Sheep and goat	Raw, thermally and process milk, milk mixtures and cheeses	12S rRNA	0.1% (cow’s milk in sheep’s and goat’s milk)	[99]
	Goat	Dairy products	12S rRNA	0.1% (goat’s milk in sheep’s milk)	[100]
	Cow and buffalo	Mozzarella cheese	12S rRNA	0.1% (cow’s milk in mozzarella cheeses)	[101]
	Goat, sheep and cow	Goat’s and sheep’s cheeses	cytb	1% (cow’s milk in goat’s cheeses)	[102]
	Goat and ovine	Ovine cheeses	12S rRNA	1% (goat’s milk in sheep’s cheeses)	[103]
	Cow, goat and sheep	Cheeses and other dairy products	12S rRNA	1% (cow’s milk in cheeses)	[37]
	Cow, sheep, goat and buffalo	Raw and pasteurized milks and cheese	k-casein	0.1% (cow’s milk in buffalo’s milk)	[104]
Multiplex PCR	Cow, goat and sheep	Mixture cheeses	12S rRNA (cow, sheep and goat) and 16S rRNA (sheep)	0.125 ng (DNA from the three species) 0.5% (cow’s milk in goat’s milk)	[105]
	Cow and sheep	Ovine cheeses	12S rRNA (cow, sheep) and 16S rRNA (sheep)	0.1% (cow’s milk in ovine cheeses)	[106]
	Cow and goat	Goat cheeses	12S rRNA	0.1% (cow’s milk in goat’s cheese)	[107]
	Cow and yak	Raw, pasteurized, and sterilized milk mixtures	12S rRNA	0.1% (cow’s milk in yak’s milk)	[108]
	Cow and buffalo	Raw and heat treated milks and cheeses	D-Loop	0.1% (both species in milk and cheese) 0.15 ng of buffalo’s and 0.04 ng cow’s DNA).	[109]
	Cow, goat, sheep and water buffalo	Dairy products (butter, cheese, cottage cheese, cream, milk (fresh, UHT, powdered) and yogurt	mtDNA	1% (in two-species milk mixtures)	[110]
	Cow and goat	Goat’s milk	mtDNA	0.5% (cow’s milk)	[111]
	Goat and cow	Goat’s cheese	12S rRNA	0.5% (cow’s milk in goat cheeses)	[112]
	Cow, sheep and goat	Mono-species Sicilian dairy products	12S rRNA (cow, goat) 12S rRNA and 16S rRNA (sheep)	0.1% (milk all species in cheeses)	[113]
	Cow, sheep and goat	Goat’s milk products (aged cheese, fresh cheese, yogurt, UHT milk and powder milk)	12S rRNA (cow and goat) and cytb (sheep)	0.05 ng (DNA of each species)	[114]
	Cow and goat	Milk powder	12S rRNA	0.1% (cow’s milk in goat’s milk)	[115]
	Cow, camel, horse and goat	Raw, freeze-dried, pasteurized and ultra-high temperature (UHT) milk	16S rRNA (camel and cow) and D-Loop (horse and goat)	0.1%, 0.2% and 0.5% (cow’s milk in raw milk and freeze-dried milk mixtures, pasteurized milk and UHT milk, respectively)	[116]
	Cow, sheep and goat	PDO Portuguese cheeses	cytb	- ^a^	[117]
Real-time PCR—SYBR Green dye	Cow and buffalo	Mozzarella cheeses	cytb	0.1% (cow’s milk)	[118]
	Cow and goat	UHT goat’s milk	12S rRNA	0.5% (cow’s milk)	[119]
	Cow, sheep and goat	Goat’s milk products (aged cheese, fresh cheese, yogurt, UHT milk and powder milk)	12S rRNA (cow and goat) and cytb (sheep)	0.005 ng (DNA of each species)	[114]
	Cow and buffalo	buffalo yogurt	cytb	0.015 ng of DNA for both species	[120]
Multiplex real-time PCR—SYBR Green dye	Cow, sheep, goat and buffalo	Milk mixtures and cheeses	12S rRNA (cow and goat) and cytb (sheep and buffalo)	0.1% (all species)	[121]
Real-time PCR—TaqMan probes	Goat and sheep	Raw and heat-treated milk mixtures	12S rRNA	0.5% (goat’s DNA) 0.6% (goat’s milk in raw and pasteurized mixtures)	[122]
	Cow and sheep	Raw and heat-treated milk mixtures	12S rRNA	0.5% (cow’s milk in raw and pasteurized sheep’s milk)	[123]
	Cow	Fresh and processed meats, milks and cheeses	cytb	35 pg cow’s DNA	[124]
	Bovine and buffalo	Cheese samples	cytb	2% (cow’s milk in buffalo’s milk)	[125]
	Cow and donkey	Raw, pasteurized and autoclaved milks	*COI*	2% (cow’s milk in donkey’s milk)	[126]
	Bovine and buffalo	Dairy products and meat	cytb (cow) and 16S rRNA (buffalo)	1% (cow’s milk in buffalo cheese)	[127]
	Cow, goat, sheep and buffalo	Dairy products	12S rRNA	≤25 ng (DNA of all species)	[128]
	Cow and goat	Milk powder	12S rRNA	0.1% (cow’s milk in goat’s milk)	[115]
	Camel	Milk mixtures	Heart development protein with EGF-like domain 1 (HEG1) (camel) Myostatin (mammalian species)	1% (camel’s milk in cow’s milk)	[129]
Multiplex real-time PCR—TaqMan probes	Cow and buffalo	milk	cytb	1% (cow DNA in buffalo DNA and vice versa)	[130]
	Cow, goat, sheep and buffalo	Milk and cheeses	Allmilk: tRNA-Lys (cow), cytb (goat, sheep and buffalo) Allcheese: β-actine (cow), prolactic receptor (sheep), grwoth hormone receptor (goat)	0.32–32 ng of DNA of all species (Allmilk)	[131]
	Cow and mare	Dairy products	12S rRNA	0.001 ng (DNA of cow milk, yogurt, and mare milk) 0.005 ng (DNA of sour soup and Koumiss)	[132]
	Cow and goat	Dairy and meat products	12S rRNA	0.005 ng and 0.01 ng (DNA of goat’s milk and cheese, respectively) 0.01 ng and 0.05 ng (DNA of cow’s milk and cheese, respectively)	[133]
	Sheep and goat	Dairy and meat products	12S rRNA	0.001 ng and 0.01 ng (DNA of fresh and processed ovine meats, respectively) 0.00025 ng, 0.005 ng and 0.01 ng (DNA of caprine meat, milk and cheese, respectively)	[134]
	Camel and cow	Dairy and meat products	12S rRNA	1% (camel and cow milk in milk mixtures) 0.005–0.0025 ng (DNA of camel milk) 0.05–0.001 ng (DNA of camel yogurt) 0.001–0.0005 ng (DNA of camel milk beverage), 0.00025–0.0001 ng (DNA of camel meat), 0.0025–0.001 ng (DNA of cow milk), 0.5–0.001 ng (DNA of cow yogurt), 1–0.05 ng (DNA of cow cheese), 0.01 ng (DNA of cow acidic whey), 0.001 ng (DNA of cow milk powder), 0.0005–0.00025 ng (DNA of beef and beef jerky), 0.005 ng (DNA of beef sausage)	[135]
HRM analysis	Cow, sheep and goat	Cheeses	D-loop	0.1% (cow’s milk in mixed-milk)	[136]
	Cow and buffalo	Buffalo dairy products	12S rRNA and D-loop	1% (cow’s milk in mozzarella cheese)	[137]
ddPCR	Cow and buffalo	Mozzarella cheeses	cytb	0.1% (cow’s milk in buffalo’s milk mozzarella cheese)	[138]
LAMP	Cow and buffalo	Milk and meat mixtures	D-loop	5% (cow’s milk in buffalo’s milk)	[139]
	Cow and goat	Milk and yogurt	cytb	2% (cow’s and goat’s milk)	[140]
NGS	Goat, sheep, cow and buffalo	Milk mixtures and cheeses	12S and 16S rRNA	-^a^	[141]
DNA biochip (microarray) kit	Cow, pig, horse, donkey, sheep, goat, water buffalo, hare, rabbit, deer, chicken, turkey, ostrich, cat, and dog	Milk and meat mixtures, and dairy and meat products	16S rRNA	0.1% (Cow’s, goat’s and buffalo’s milk)	[142]
DNA hybridization on microspheres	Cow, sheep and goat	Milk mixtures and yogurts	cytb	0.01% (cow’s milk in goat’s yogurt and 0.05% (cow’s milk in sheep’s yogurt)	[143]
Paper-based DNA biosensor	Cow, sheep and goat	Milk mixture yogurts	cytb (cow and sheep) and prolactic receptor (sheep),	0.01% of cow’s yogurt	[144]

ddPCR, droplet digital PCR; HRM, high-resolution melting; LAMP, loop-mediated isothermal amplification; NGS, next generation sequencing; SSR, simple sequence repeats; ^a^ not reported.

**Table 4:** Summary of pros and cons of the main techniques applied for species identification in dairy products.

Technique	Pros	Cons
Electrophoretic techniques	Fast Low cost	Complex band pattern or co-migrating bands can lead to an equivocal interpretation of results Inadequate for quantification, processed products and/or detecting adulteration by heat-treated bovine whey protein Need of reference standards (IEF)
Immunochemical techniques	Fast Simple -Low cost High sensitivity Easy application in routine analysis	Possible cross-reactivity leading to false positives Processing might lead to false negative results Availability of specific antibodies
Chromatography coupled to mass spectrometry	High specificity High sensitivity Quantitative Possibility of multiplex	Costly equipment and maintenance Complex analysis Requires databases Highly expertise technicians
Spectroscopy	Fast Simple High-throughput Non-destructive Capacity of portability (depending on the technique)	Requires a large database and chemometrics Expensive equipment (depending on the technique)
PCR-RFLP	Simple High specificity	Not quantitative
Species-specific PCR	Simple High sensitivity Possibility of multiplex	Not quantitative
Real-time PCR	High sensitivity High specificity Quantitative Possibility of multiplex Fast	Moderate cost of equipment
Biosensors	Fast User-friendly Low-cost High-throughput Potential of portability	Qualitative results Sensitivity can be low
